# Hearing Loss and Oxidative Stress: A Comprehensive Review

**DOI:** 10.3390/antiox13070842

**Published:** 2024-07-14

**Authors:** A. Maniaci, L. La Via, J. R. Lechien, G. Sangiorgio, G. Iannella, G. Magliulo, A. Pace, Q. Mat, S. Lavalle, M. Lentini

**Affiliations:** 1 Faculty of Medicine and Surgery, University of Enna Kore, 94100 Enna, Italy; salvatore.lavalle@unikore.it; 2 ASP Ragusa-Hospital Giovanni Paolo II, 97100 Ragusa, Italy; marlentini@tiscali.it; 3 Otology Study Group, Young Otolaryngologists-International Federation of Otorhinolaryngological Societies, 13005 Paris, France; jerome.lechien@umons.ac.be (J.R.L.); giannicola.iannella@uniroma1.it (G.I.); annalisa.pace@uniroma1.it (A.P.); quentin.mat@chu-charleroi.be (Q.M.); 4 Department of Anaesthesia and Intensive Care, University Hospital Policlinico-San Marco, 95125 Catania, Italy; 5 Department of Human Anatomy and Experimental Oncology, Faculty of Medicine, UMONS Research Institute for Health Sciences and Technology, University of Mons (UMons), 7000 Mons, Belgium; 6 Department of Otolaryngology-Head & Neck Surgery, Foch Hospital, School of Medicine, UFR Simone Veil, Université Versailles Saint-Quentin-en-Yvelines (Paris Saclay University), 78180 Paris, France; 7 Department of Otolaryngology-Head & Neck Surgery, EpiCURA Hospital, 7301 Hornu, Belgium; 8 Department of Biomedical and Biotechnological Sciences, University of Catania, 95123 Catania, Italy; giuseppe.sangiorgio@phd.unict.it; 9 Department of ‘Organi di Senso’, University “Sapienza”, 00185 Rome, Italy; giuseppe.magliulo@uniroma1.it; 10 Department of Otorhinolaryngology, C.H.U. Charleroi, Chaussée de Bruxelles 140, 6042 Charleroi, Belgium

**Keywords:** hearing loss, oxidative stress, antioxidants, gene therapy, inflammation

## Abstract

Hearing loss is a prevalent condition affecting millions of people worldwide. Hearing loss has been linked to oxidative stress as a major factor in its onset and progression. The goal of this thorough analysis is to investigate the connection between oxidative stress and hearing loss, with an emphasis on the underlying mechanisms and possible treatments. The review addressed the many forms of hearing loss, the role of reactive oxygen species (ROS) in causing damage to the cochlea, and the auditory system’s antioxidant defensive mechanisms. The review also goes over the available data that support the use of antioxidants and other methods to lessen hearing loss brought on by oxidative stress. We found that oxidative stress is implicated in multiple types of hearing loss, including age-related, noise-induced, and ototoxic hearing impairment. The cochlea’s unique anatomical and physiological characteristics, such as high metabolic activity and limited blood supply, make it particularly susceptible to oxidative damage. Antioxidant therapies have shown promising results in both animal models and clinical studies for preventing and mitigating hearing loss. Emerging therapeutic approaches, including targeted drug delivery systems and gene therapy, offer new possibilities for addressing oxidative stress in the auditory system. The significance of this review lies in its comprehensive analysis of the intricate relationship between oxidative stress and hearing loss. By synthesizing current knowledge and identifying gaps in understanding, this review provides valuable insights for both researchers and clinicians. It highlights the potential of antioxidant-based interventions and emphasizes the need for further research into personalized treatment strategies. Our findings on oxidative stress mechanisms may also affect clinical practice and future research directions. This review serves as a foundation for developing novel therapeutic approaches and may inform evidence-based strategies for the prevention and treatment of hearing loss, ultimately contributing to improved quality of life for millions affected by this condition worldwide.

## 1. Introduction

Over 1.5 billion individuals globally suffer from hearing loss, and estimates suggest that number will rise to 2.5 billion by 2050 [1]. Beyond only making it difficult to hear, hearing loss can have a significant negative influence on a person’s quality of life and cause social isolation, depression, and cognitive deterioration [2,3]. Although several variables contribute to the development of hearing loss, oxidative stress has been identified as a key role in the pathophysiology of hearing loss [4]. When the body’s capacity to eliminate reactive oxygen species (ROS) through antioxidant defenses is out of balance, oxidative stress occurs [5]. Cellular damage, malfunction, and finally cell death are the outcomes of this imbalance [6]. Numerous illnesses, such as cancer, neurological diseases, and cardiovascular disorders, have been linked to oxidative stress [7,8]. Studies have shown that oxidative stress has detrimental effects on the auditory system and is linked to age-related hearing loss, noise-induced hearing loss, and ototoxicity [9,10]. Because of its high metabolic requirements and exposure to a variety of external stressors, including noise and ototoxic medications, the auditory system is particularly susceptible to oxidative stress [9]. Hearing loss is mostly caused by oxidative damage to the inner ear, specifically to the cochlea [11]. Sound waves must be converted into electrical signals by sensory hair cells, which are found in the highly specialized cochlea [12]. Due to their low ability to regenerate, these hair cells are especially vulnerable to oxidative damage [13]. Research has repeatedly demonstrated that oxidative stress markers are more prevalent in hearing-impaired people than in normal-hearing people [14,15]. In comparison to healthy controls, individuals with sensorineural hearing loss had far greater amounts of malondialdehyde, a marker of lipid peroxidation, according to a study by Karlidag et al. [16]. In a similar vein, Neri et al. [17] showed that patients with age-related hearing loss exhibited decreased antioxidant enzyme activity and elevated levels of oxidative stress indicators. Considering the significant effects that hearing loss has on people and society at large, it is imperative to comprehend the connection between oxidative stress and hearing loss in order to create preventative and therapeutic measures that will work. The objectives of this thorough study are to investigate the mechanisms that lead to hearing loss caused by oxidative stress, the protective effect of antioxidants against this damage, and possible future paths for clinical research and treatment. Examining the causes of reactive oxygen species (ROS) in the auditory system, as well as the antioxidant defense mechanisms that prevent them, is essential. Additionally, it is important to explore the specific pathways through which oxidative stress results in hearing loss, such as inflammation, ischemia–reperfusion injury, hair cell death, and mitochondrial dysfunction. The potential of antioxidant therapies, including pharmaceutical drugs, gene therapy, and dietary antioxidants, in the prevention and treatment of hearing loss will also be covered in this review. Although there have been several evaluations on the connection between oxidative stress and hearing loss, our work makes several unique contributions to the area. This review offers a comprehensive understanding of oxidative stress in hearing loss by synthesizing recent developments from several fields, including audiology, otolaryngology, biochemistry, and molecular biology. We extend the body of research by providing a thorough examination of novel interventions not fully addressed in earlier reviews, such as gene therapy, stem cell treatments, and drug delivery methods based on nanotechnology. By bridging the gap between fundamental research and practical applications, our method offers guidance on how current discoveries in oxidative stress processes can impact clinical practice and direct future treatment approaches. We evaluate the field’s present research approaches objectively, noting their advantages, disadvantages, and potential areas of development for further research. By conducting this critical evaluation, we are able to pinpoint important knowledge gaps and suggest particular topics for further study, which may serve as a roadmap for the subsequent round of studies in this area. Additionally, we provide a distinct viewpoint on how the knowledge of oxidative stress in hearing loss might be converted into useful clinical guidelines for treatment, early intervention, and prevention. Our review differs from the previous literature due to its translational focus, which makes it beneficial for researchers and clinicians alike. By addressing these facets, this study offers a forward-looking perspective on the function of oxidative stress in hearing loss while also consolidating present knowledge. Our goal is to provide a thorough, insightful, and clinically applicable analysis that sets our work apart from previous evaluations and makes a significant contribution to the field’s advancement.

## 2. Methods

Several electronic databases, including PubMed, Scopus, Web of Science, and Google Scholar, were used to perform an extensive literature search. Several key terms were used in the search strategy, including “hearing loss”, “oxidative stress”, “reactive oxygen species”, “antioxidants”, “cochlea”, “hair cells”, “mitochondria”, “inflammation”, “ischemia-reperfusion”, “noise-induced hearing loss”, “age-related hearing loss”, and “ototoxicity”. To guarantee that pertinent research was included in the search results, boolean operators (AND, OR) were employed. No year or language restriction was applied in the search. In order to find any more pertinent research that might have gone unnoticed during the original database search, the reference lists of the included papers were additionally manually searched. We included the following. 1. Original research papers, reviews, and meta-analyses that looked into the connection between hearing loss and oxidative stress. 2. Research that investigated the pathways—such as mitochondrial malfunction, ischemia–reperfusion injury, inflammation, and hair cell death—that underlie oxidative stress-induced hearing loss. 3. Research on the use of pharmaceutical drugs, gene therapy, and nutritional antioxidants as antioxidant therapies to cure and prevent hearing loss. 4. Studies involving animals, in vitro subjects, and humans were all taken into consideration for inclusion. We excluded the following. 1. Conference abstracts, opinions, case reports, and letters to the editor. 2. Research that did not concentrate on the connection between hearing loss and oxidative stress. 3. Research that was not released in English. The relevance of the titles and abstracts of the identified publications was checked by two separate reviewers. After that, full-text publications for the chosen studies were obtained, and their eligibility was further evaluated in accordance with the inclusion and exclusion criteria. After the data were retrieved, a narrative approach was used to synthesize the information, with an emphasis on the mechanisms underlying hearing loss caused by oxidative stress and the potential benefits of antioxidant therapies for both prevention and treatment of this illness. To provide a more nuanced view of the problem, subgroup analyses were conducted based on the kind of hearing loss (e.g., age related, noise induced, ototoxicity) and the specific antioxidant intervention (e.g., dietary antioxidants, pharmaceutical agents, gene therapy). Following a thorough analysis, the results were presented in relation to the state of the literature, emphasizing the main conclusions, restrictions, and implications for further study and clinical application. In order to close these information gaps and progress the topic of oxidative stress and hearing loss, the review also suggested future research directions.

## 3. Results

**Figure 1:**
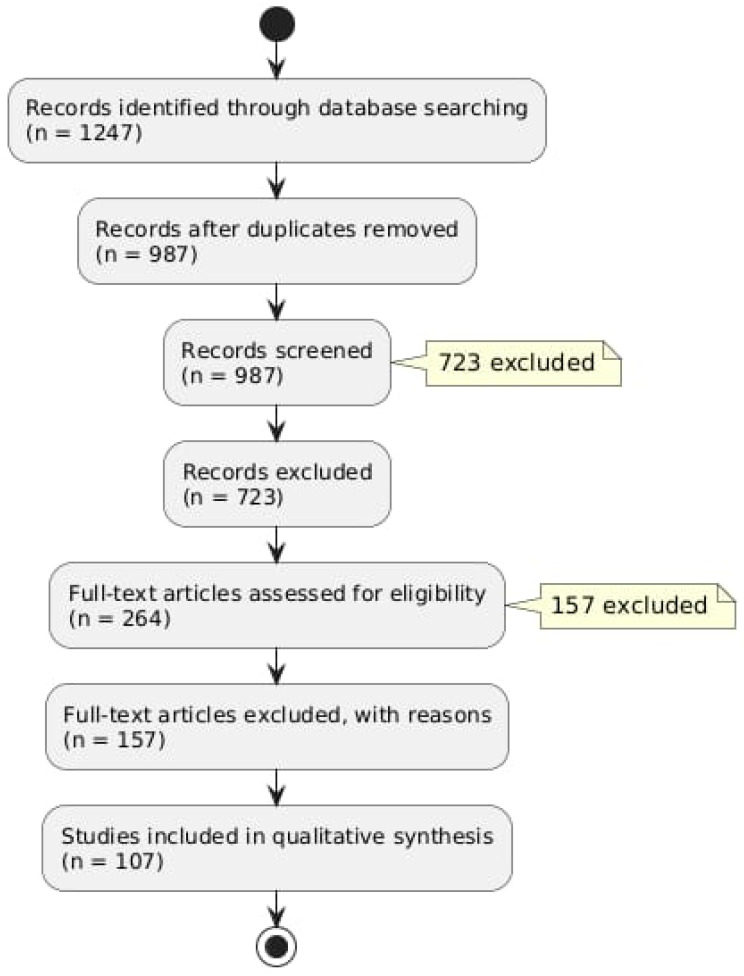
Flow diagram describing the literature research protocol.

A preliminary search of the database produced 1247 records. There were 987 unique records left after duplicates were eliminated. A total of 723 records that did not fit our inclusion criteria were excluded as a result of title and abstract screening. Figure 1 provides a summary of our literature search findings (Figure 1). 

After evaluating 264 full-text articles for eligibility, 156 of them were disqualified for a variety of reasons, such as publications written in languages other than English or a lack of emphasis on the connection between oxidative stress and hearing loss. There were 108 studies in the final review.

## 4. How Are Oxidative Stress and the Auditory System Related?

Reactive oxygen species’ (ROS) generation and the body’s capacity to counteract them through antioxidant defenses are out of balance in oxidative stress [18]. ROS are very reactive substances that have the ability to harm lipids, proteins, and DNA, among other cellular constituents, ultimately resulting in cellular malfunction and demise [19]. NADPH oxidases, xanthine oxidase, and the mitochondrial electron transport chain are the main producers of reactive oxygen species (ROS) [20]. Even while ROS are physiologically significant for the immune system and cell signaling, excessive ROS generation can cause oxidative stress and aid in the etiology of many disorders, including hearing loss [21]. The cochlea, in particular, is extremely vulnerable to oxidative stress because of the special anatomical and physiological traits of the auditory system [22]. There are different primary sources of ROS in the auditory system. The increased ROS generation and mitochondrial activity are the results of the high metabolic needs of the cochlea, particularly in the sensory hair cells and stria vascularis [23]. Via a number of pathways, including NADPH oxidase activation, glutamate excitotoxicity, and mitochondrial dysfunction, exposure to high noise levels can increase the production of reactive oxygen species (ROS) in the cochlea [9,24]. By raising ROS production and lowering antioxidant defenses, some pharmaceuticals, including cisplatin and aminoglycoside antibiotics, can cause oxidative stress in the cochlea [25,26]. Decreased antioxidant capacity and mitochondrial dysfunction in the cochlea are age-related alterations that lead to oxidative stress and hearing loss [4]. The auditory system is equipped with a sophisticated network of antioxidant defense mechanisms to mitigate the harmful effects of reactive oxygen species [27]. The main enzymes that neutralize ROS and shield the cochlea from oxidative damage are glutathione peroxidase (GPx), catalase (CAT), and superoxide dismutase (SOD) [28]. Glutathione (GSH), vitamins C and E, and coenzyme Q10 are examples of small-molecule antioxidants that are essential for scavenging reactive oxygen species (ROS) and preserving redox balance in the cochlea [29,30]. The cochlea’s cellular redox equilibrium is preserved by the nuclear factor erythroid 2-related factor 2 (Nrf2) pathway, which is a key regulator of antioxidant gene expression [30]. Oxidative stress occurs when the generation of ROS surpasses the antioxidant defenses of the auditory system, resulting in a variety of pathological alterations in the cochlea [4]. Direct damage from oxidative stress to sensory hair cells can result in their malfunction and eventual death, which is the main cause of hearing loss [31]. Hearing loss can be made worse by a vicious loop of increased ROS generation and energy depletion brought on by damage to mitochondrial DNA, proteins, and lipids caused by ROS [22]. Oxidative stress has the ability to trigger inflammatory pathways within the cochlea. Oxidative stress in the cochlea can trigger an inflammatory response, leading to the activation and recruitment of immune cells. This process involves the activation of resident immune cells as macrophages and leukocytes in the cochlea and the upregulation of pro-inflammatory cytokines, such as TNF-α, IL-1β, and IL-6, and increased vascular permeability. [32]. Activated immune cells can also produce more ROS, creating a feedback loop that further exacerbates cochlear damage. The stria vascularis, which is in charge of preserving the endocochlear potential, is especially susceptible to oxidative stress, and when it malfunctions, hearing loss may result [32].

## 5. What Are the Mechanisms of Hearing Loss Induced by Oxidative Stress?

**Figure 2:**
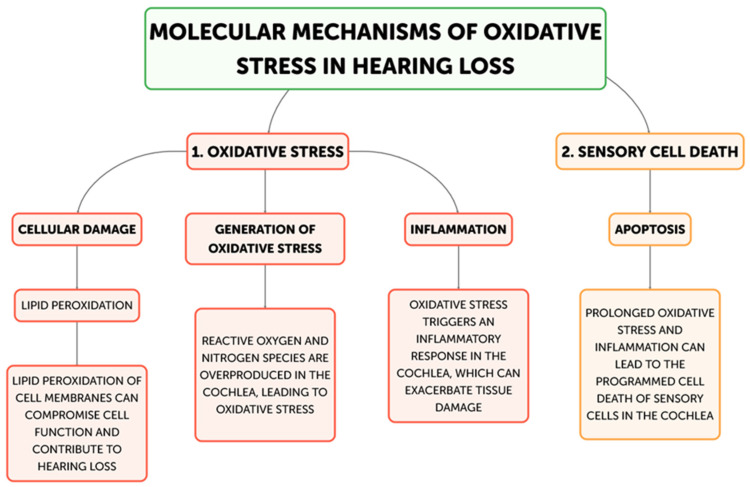
Flow diagram for molecular mechanisms of Oxidative stress related to Hearing loss development.

Hearing loss is primarily caused by oxidative stress, and there are multiple ways that excessive generation of reactive oxygen species (ROS) can harm the auditory system. The main mechanisms underpinning oxidative stress-induced hearing loss will be discussed in this part, including inflammation, ischemia–reperfusion injury, hair cell death, and mitochondrial dysfunction. The main places in cells where reactive oxygen species (ROS) are produced are the mitochondria, and oxidative stress-induced hearing loss is largely caused by dysfunctional mitochondria [21] (Figure 2).

**Figure 3:**
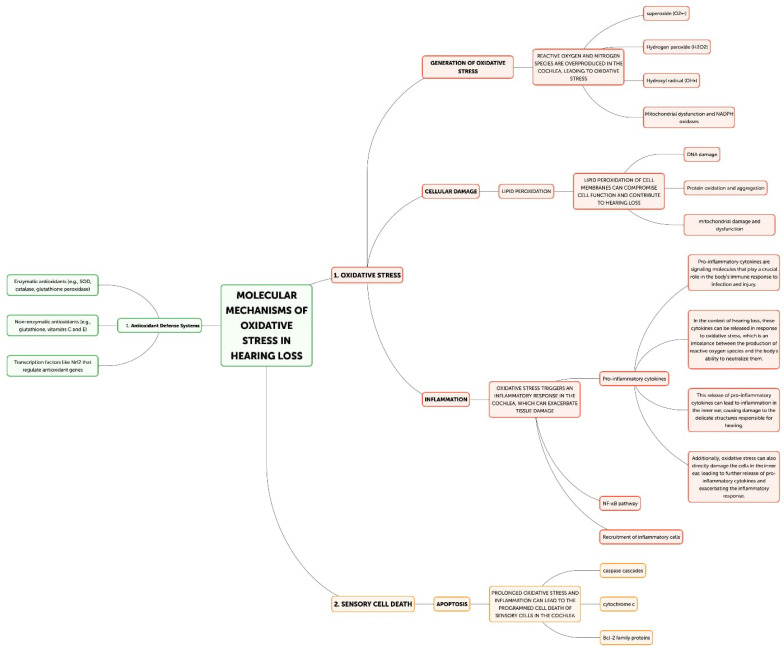
Molecular mechanism of oxidative stress in hearing loss.

The cochlea depends heavily on mitochondrial function for energy production due to its high metabolic needs, especially in the sensory hair cells and stria vascularis [23]. On the other hand, excessive ROS production can harm proteins, lipids, and mitochondrial DNA, which impairs mitochondrial activity and produces more ROS [22]. Superoxide (O_2_^•−^) is frequently the first reactive oxygen species (ROS) to develop, mostly as a result of NADPH oxidase activation or mitochondrial electron transport chain leakage. The cochlea’s unique NADPH oxidase, NOX3, is a significant generator of superoxide. NOX3 produces a lot of superoxides when it is activated by ototoxic stimuli or noise stress, which starts a chain reaction of oxidative processes. Iron–sulfur clusters in proteins can be directly damaged by superoxide, which can result in the release of free iron and the inactivation of enzymes, exacerbating oxidative stress. Superoxide dismutates to create hydrogen peroxide (H_2_O_2_), which is less reactive than superoxide but more persistent and diffusible. H_2_O_2_ can cross membranes in the cochlea and oxidize different parts of the cell. It is important for redox signaling because it affects transcription factors, like NF-κB and AP-1, which control cochlear cell apoptotic and inflammatory responses. Furthermore, Fenton reactions involving excess H_2_O_2_ and transition metals can produce extremely corrosive hydroxyl radicals. The most reactive ROS, hydroxyl radicals (OH^•^), destroy cellular macromolecules without discrimination. The lipid-rich outer hair cell membranes in the cochlea are especially vulnerable to the damaging effects of hydroxyl radicals, which can cause lipid peroxidation cascades that impair membrane integrity and cellular function [33]. This process is particularly important in noise-induced hearing loss because it can quickly cause the death of outer hair cells through the acute production of hydroxyl radicals. In the end, the auditory system may experience energy depletion, cellular malfunction, and cell death as a result of this destructive cycle of mitochondrial damage and ROS generation [34]. Research has demonstrated a correlation between age-related hearing loss and mutations and deletions in the mitochondrial DNA, underscoring the significance of mitochondrial function in preserving auditory health [35,36]. The loss of sensory hair cells in the cochlea, which are especially susceptible to oxidative stress, is the main cause of hearing loss [31]. Both necrotic and apoptotic mechanisms can cause hair cell death when exposed to oxidative stress [37]. Cell enlargement, organelle malfunction, and rupture of the plasma membrane—which results in the release of cellular contents and inflammation—are the passive processes that define necrosis [38]. On the other hand, apoptosis is a route of programmed cell death that includes chromatin condensation, caspase activation, and the creation of apoptotic bodies [39]. By triggering caspases, releasing cytochrome c, and activating mitochondrial permeability transition pores (mPTPs), ROS can cause apoptosis in hair cells [40]. Furthermore, oxidative stress can trigger additional apoptotic pathways, including the JNK and p53 signaling cascades, which can further exacerbate the death of hair cells [41,42]. Hearing loss can result from processes that are intimately related to oxidative stress and inflammation (Figure 3) [32]. Pro-inflammatory cytokines, chemokines, and adhesion molecules can be produced as a result of ROS-activating inflammatory pathways, such as the NF-κB and MAPK signaling cascades [43]. These inflammatory mediators have the ability to draw in and activate immune cells, like neutrophils and macrophages, which can worsen tissue damage and oxidative stress in the cochlea [44]. Furthermore, hair cell loss and auditory impairment can be exacerbated by the activation of resident immune cells in the cochlea, such as fibrocytes and macrophages, which can prolong the inflammatory response [45,46]. The etiology of age-related hearing loss has also been linked to inflammaging, a chronic low-grade inflammatory response linked to aging [47]. Another way oxidative stress might cause hearing loss is by ischemia–reperfusion damage [48]. Because the cochlea is so sensitive to changes in blood flow, ischemia during the reperfusion period might cause ROS to be produced [49]. Hair cells, spiral ganglion neurons, and other cochlear structures may sustain oxidative damage as a result of this surge in ROS generation, which may outweigh the cochlea’s antioxidant defenses [50]. An important modulator of cellular antioxidant responses is the nuclear factor erythroid 2-related factor 2 (Nrf2) pathway. Nrf2 translocates to the nucleus in response to oxidative stress, where it binds to Antioxidant Response Elements (AREs) and upregulates the expression of genes related to glutathione production and other antioxidant enzymes. Chronic oxidative stress, however, has the potential to overpower or compromise this defense system. Poly(ADP-ribose) polymerase-1 (PARP-1) is activated by ROS-induced DNA damage, especially in mitochondrial DNA. Although PARP-1 aids in DNA repair, overactivation of the protein can cause cochlear cells to experience an energy crisis and NAD+ depletion, which ultimately leads to cell death. ROS cause lipid peroxidation in the membranes of cochlear cells, especially influencing the outer hair cells that are rich in phospholipids. Reactive aldehydes, like 4-hydroxynonenal (4-HNE), are produced by this mechanism, and they have the ability to create protein adducts and spread cellular harm. Oxidative stress in the cochlea can set off an inflammatory response that activates leukocytes and macrophages, two types of native immune cells. Pro-inflammatory cytokines such TNF-α, IL-1β, and IL-6 are upregulated as a result [7]. These cytokines can worsen the harm that oxidative stress causes to the cochlea and are known to have a role in the inflammatory process [8]. Furthermore, increased production of reactive oxygen species (ROS) by the activated immune cells might result in a feedback loop that exacerbates cochlear damage [32]. The article also notes that transcription factors, like NF-κB and AP-1, which control inflammatory and apoptotic responses in cochlear cells, can be activated by hydrogen peroxide (H_2_O_2_), a reactive oxygen species [34]. Additionally, oxidative stress has the ability to initiate apoptotic pathways, including the JNK and p53 signaling cascades, which may contribute to the cochlea’s hair cells dying [41,42].

Antioxidant therapy can mitigate the considerable hair cell loss and auditory impairment that ischemia–reperfusion injury can induce in animals [9,50]. Ischemia–reperfusion damage and oxidative stress have been linked to cochlear blood flow impairment in humans, including presbycusis and abrupt sensorineural hearing loss [4,51].

## 6. Different Types of Hearing Loss and Oxidative Stress: Pathophysiological Mechanisms

It is essential to investigate the role that oxidative mechanisms play in different forms of auditory impairment in order to improve our comprehension of the intricate link between oxidative stress and hearing loss. This method gives insights into possible focused treatments in addition to a thorough framework. Oxidative stress has a complex involvement in presbycusis, an age-related hearing loss [2,28]. Different cochlear structures are impacted by the slow accumulation of oxidative damage over time [24]. For example, reduced endocochlear potential caused by mitochondrial malfunction in the stria vascularis impairs the cochlea’s capacity to transduce sound [4]. Hair cells have a build-up of mutations in their mitochondrial DNA at the same time, which eventually sets off apoptotic pathways. The oxidative stress-induced production of advanced glycation end products (AGEs) exacerbates the situation by decreasing the compliance of the tectorial and basilar membranes, which are crucial for hearing [14,15]. An alternative scenario is that of noise-induced hearing loss, in which the cochlea experiences an acute increase in reactive oxygen species (ROS) generation that occurs quickly and intensely [11]. Widespread lipid peroxidation results from this abrupt oxidative burst, which especially damages the fragile outer hair cells. The apoptotic cascades are triggered by the release of cytochrome c, and the ischemia–reperfusion injury caused by noise-induced vasoconstriction in the stria vascularis intensifies the damage [27,34]. Different oxidative stress processes are involved in ototoxicity-induced hearing loss, which is frequently linked to specific drugs. For instance, iron and aminoglycoside antibiotics combine to produce complexes that catalyze the production of ROS [27]. On the other hand, chemotherapeutics based on platinum, such as cisplatin, deplete antioxidant systems and activate NOX3, which is a major source of reactive oxygen species in the cochlea [28,43]. Both paths converge on spiral ganglion neuron degeneration and hair cell death, despite their distinct beginning processes. Although complex in nature, sudden sensorineural hearing loss commonly involves acute oxidative stress. The early start of oxidative damage, whether due to viral infections, vascular events, or autoimmune reactions, can swiftly overwhelm local antioxidant defenses, leading to rapid and severe hearing impairment [46]. Hearing loss caused by oxidative stress is also influenced by genetic factors. Genes such as GJB2, which encodes connexin 26, can be mutated to affect important functions, such as potassium recycling, which can result in an increased production of ROS and metabolic stress [24]. Similar to this, in cochlear cells, mutations that impair mitochondrial activity can have a direct effect on cellular energy production and ROS control [23,25]. Gaining knowledge of these many pathophysiological processes is essential for creating focused treatment plans. Therapies targeted at promoting mitochondrial activity may be very helpful in several disorders [52,53], in particular age-related hearing loss, and fast-acting antioxidants may be essential in noise- or abrupt-induced hearing loss [54]. Strategies that strengthen endogenous antioxidant systems or directly block particular ROS-generating pathways may work well in cases of ototoxicity. Furthermore, tailored therapy methods have new opportunities thanks to this sophisticated understanding of oxidative stress in various forms of hearing loss. Through the identification of the major oxidative pathways involved in individual cases, healthcare providers may be able to more effectively customize prevention and treatment plans. In cases where there are known genetic components, this may entail a mix of systemic and local antioxidant medications, lifestyle changes to lessen the oxidative burden, or even genetic therapies that target certain pathways [55]. By delving into the particular pathophysiological pathways of oxidative stress in different kinds of hearing loss, we further advance scientific knowledge and open the door to more focused, successful treatment interventions. This strategy fills the knowledge gap between fundamental science and practical application by providing a thorough framework that can direct future investigations and influence therapeutic procedures in the field of auditory health.

## 7. How Can Antioxidant Techniques Prevent and Treat Hearing Loss?

Antioxidant-based interventions have emerged as viable approaches to prevent and treat hearing loss due to the essential role that oxidative stress plays in the etiology of this disorder. This section will examine several antioxidant strategies that have demonstrated promise in reducing oxidative stress-induced hearing loss, such as pharmaceutical treatments, targeted delivery methods, gene therapy, and dietary adjustments. The potential of dietary antioxidants to prevent and treat hearing loss has been the subject of much research [52]. These antioxidants include polyphenols, like quercetin, curcumin, and resveratrol, as well as vitamins A, C, and E [53,54] (Table 1). By lowering oxidative stress and inflammation in the cochlea, dietary supplements containing these antioxidants have been demonstrated in animal studies to mitigate age- and noise-related hearing loss [55,56]. Higher dietary antioxidant intake has been linked to a lower risk of hearing loss in people, according to observational studies [57,58]. Nevertheless, to determine if dietary antioxidants are effective in both preventing and treating human hearing loss, randomized controlled trials are required. The potential of pharmacological antioxidants to lessen hearing loss caused by oxidative stress has also been studied [59]. N-acetylcysteine (NAC), ebselen, D-methionine, and coenzyme Q10 (CoQ10) are some of these agents [60,61]. By scavenging reactive oxygen species (ROS) and bolstering endogenous antioxidant defenses, NAC, a precursor of glutathione, has been demonstrated to protect against noise-induced and ototoxic drug-induced hearing loss in animal models [62,63]. In a similar vein, it has been discovered that the mitochondrial antioxidant CoQ10 can reduce age-related hearing loss in both people and animals [64,65]. In preclinical studies, D-methionine and ebselen have also shown protective benefits against ototoxic drug-induced hearing loss and noise-induced hearing loss [66,67]. Even though these pharmacological antioxidants seem promising, more clinical research is required to determine their efficacy and safety in people. Antioxidants may be delivered to the inner ear specifically to maximize therapeutic benefit and reduce systemic negative effects [68]. Antioxidants like resveratrol and NAC have been delivered straight to the cochlea using nanoparticle-based delivery methods, including liposomes and polymeric nanoparticles [69,70]. When compared to a systemic injection, these targeted delivery strategies have demonstrated increased efficacy in attenuating age- and noise-induced hearing loss in animal models [71,72]. Another intriguing method for adjusting the inner ear’s antioxidant defenses is gene therapy [73]. It has been possible to transfer genes encoding antioxidant enzymes, such as catalase and superoxide dismutase, to the cochlea via adenoviral and adeno-associated viral (AAV) vectors [74,75]. In animal models, these gene therapy techniques have shown preventive benefits against age-related and noise-induced hearing loss [76,77]. To optimize targeted delivery methods and gene therapy strategies for clinical translation, more studies are necessary. A comprehensive strategy to prevent and manage hearing loss caused by oxidative stress should include lifestyle changes and noise reduction techniques [78]. One of the main risk factors for hearing loss is exposure to loud noise. Noise-induced hearing impairment can be avoided by limiting noise exposure, using hearing protection devices, and avoiding loud places [79]. In addition, hearing loss risk can be decreased and total antioxidant defenses strengthened by eating a well-balanced, antioxidant-rich diet, exercising frequently, abstaining from tobacco use, and limiting alcohol intake [80,81]. In addition, sustaining auditory health requires close observation and management of long-term illnesses that might exacerbate oxidative stress and hearing loss, such as diabetes and cardiovascular disease [82,83].

Antioxidant therapies have received a lot of attention lately due to their potential to treat hearing loss brought on by oxidative stress. But, for researchers and clinicians alike, a nuanced understanding of their limitations, accompanying obstacles, and effectiveness is essential. Antioxidants found in food, like vitamins C and E and β-carotene, have demonstrated potential in preventing age-related and noise-induced hearing loss. The evidence is still conflicting, though, with several trials showing little or no benefit. The blood–labyrinth barrier is a major obstacle to these chemicals’ bioavailability in the cochlea. One example is the low penetration of vitamin C into the fluids of the inner ear. In order to improve cochlear bioavailability, researchers are investigating novel delivery methods, such as nanoparticle compositions. Even though they are usually thought to be harmless, large dosages of some antioxidants can have negative effects. It has been difficult, in addition, to consistently translate these discoveries into clinical benefits. Like many antioxidant medications, NAC has problems with bioavailability, such as low cochlear penetration and poor oral absorption. Research on drug delivery strategies that target the cochlea may be able to get around these restrictions. Pharmaceutical antioxidants have different safety profiles. While NAC is generally well tolerated, large doses can cause allergic responses or nausea. Before beginning treatment, a thorough patient assessment is essential due to the possibility of drug interactions, such as those that may occur between NAC and specific blood pressure drugs. An interesting new frontier in the prevention and treatment of hearing loss is the use of gene therapy techniques that target antioxidant pathways in the inner ear. Positive preclinical findings suggest that cochlear cells may produce targeted antioxidants over an extended period of time. This strategy might get over established bioavailability obstacles. Nevertheless, there is a dearth of long-term safety data and scant clinical evidence on people. Thorough research is necessary to identify potential dangers, which include immunological reactions to viral vectors and inadvertent off-target effects.

The various antioxidant techniques differ greatly in their practical aspects. While gene therapy options now face substantial cost and administration issues, dietary interventions may be more accessible and cost-effective for a greater number of individuals. The practicality of different antioxidant therapies is greatly influenced by these parameters.

## 8. Combination Therapies, Synergistic Approaches, and Long-Term Outcomes

Antioxidants and anti-inflammatory medications or neurotrophic factors have been combined in recent studies to maximize therapeutic effects [88,89,90,91]. Combination therapy makes sense because of the intricate interactions that occur in the pathophysiology of hearing loss involving oxidative stress, inflammation, and cellular damage [90,91,92]. For example, when combined with corticosteroids, N-acetylcysteine (NAC) has demonstrated increased effectiveness in preventing noise-induced hearing loss as opposed to when either medication is used alone [93,94]. In an investigation conducted by Fetoni et al., this combination dramatically decreased hair cell loss and hearing threshold alterations in mice exposed to noise [2]. NAC’s antioxidant qualities work in tandem with corticosteroids’ anti-inflammatory effects to potentially offer more complete protection against cochlear damage. In preclinical research, combining antioxidants with neurotrophic factors, such as neurotrophin-3 (NT-3) or the brain-derived neurotrophic factor (BDNF), has shown encouraging outcomes. The co-administration of the BDNF and the antioxidant Trolox improved spiral ganglion neuron survival in deafened guinea pigs, according to research by Sly et al. [95]. Together, these mixtures scavenge free radicals while also encouraging cochlear hair cell survival and regeneration, thereby providing a two-pronged approach to hearing preservation and restoration. Nevertheless, creating potent combination treatments comes with a number of difficulties. It is necessary to determine the ideal dosage schedules in order to optimize synergistic effects and reduce the possibility of negative interactions. The significance of timing in combination therapy was brought to light by a study conducted by Eastwood et al., which showed that in a model of electrode insertion trauma, sequential delivery of dexamethasone and NAC was more efficacious than simultaneous delivery [4]. When it comes to age-related or chronic disorders causing hearing loss, antioxidant therapies raise serious questions about the long-term safety and effectiveness of these treatments. Since there is currently little information available in this field, patients on long-term antioxidant regimens require close observation and extensive follow-up research. Many antioxidant therapies still lack long-term safety data, especially when it comes to hearing health. Even though dietary antioxidants are usually regarded as harmless, long-term high-dose supplementation may be dangerous. As per the ATBC study, smokers’ risk of lung cancer was observed to increase when they took high-dose beta-carotene supplements for an extended period of time [5]. It is unknown if there are comparable hazards for cochlear health, but further research is necessary. Concerns regarding possible changes in redox signaling or disruption of regular physiological functions are also raised by the long-term usage of pharmacological antioxidants. Long-term disruption of the cochlea’s sensitive redox equilibrium may have unforeseen repercussions. Proper function depends on this balance. Short-term NAC treatment prevented noise-induced hearing loss, according to a study by Rousset et al., while long-term administration enhanced oxidative stress in the cochlea [6]. There is little evidence to support the long-term advantages of chronic antioxidant use in reducing age-related hearing loss or delaying the advancement of chronic hearing problems. According to a prospective study by Gopinath et al., older persons who consume more vitamin C and E in their diets are at a lower risk of developing hearing loss [7]. To demonstrate causality and identify the best intervention techniques, however, randomized controlled trials with long follow-up periods are required. The long-term safety and effectiveness of antioxidant therapy may also be impacted by population-specific characteristics. The reaction of an individual to long-term antioxidant consumption may vary depending on factors such as age, genetic makeup, and co-occurring medical disorders. The effectiveness of antioxidant supplementation in mitigating noise-induced hearing loss differed depending on glutathione S-transferase gene polymorphisms, according to a study by Hou et al. [8].

## 9. What Are the Future Directions of Hearing Loss Management?

Research and therapy options are expanding as our understanding of the mechanisms underlying oxidative stress-induced hearing loss deepens. The management of hearing loss will be examined in this section, with particular attention paid to preclinical research, clinical trials, existing constraints and difficulties, and prospective topics for more studies. The processes underlying oxidative stress-induced hearing loss have been clarified, and possible treatment targets have been identified through preclinical research utilizing in vitro systems and animal models [88]. Subsequent preclinical studies ought to concentrate on delineating the molecular underpinnings of oxidative stress in the auditory system, including the functions of antioxidant enzymes, particular ROS species, and signaling pathways [89]. Preclinical research should also prioritize the creation and improvement of tailored antioxidant delivery methods, such as hydrogels and nanoparticles [90]. Advanced methods, such as mass spectrometry imaging and single-cell RNA sequencing, can shed light on the temporal and spatial patterns of oxidative stress in the cochlea [91,92]. Moreover, the translation of preclinical discoveries to clinical applications can be aided by the creation of innovative animal models that more closely resemble situations associated with hearing loss in humans, such as age-related and noise-induced hearing loss [93]. To evaluate the safety and effectiveness of antioxidant-based treatments for hearing loss in people, clinical trials are crucial. More thorough, large-scale clinical trials are required to determine the clinical value of antioxidants, such as N-acetylcysteine (NAC) and vitamin E, even though some clinical studies have shown encouraging results in treating and preventing noise-induced hearing loss [56,94]. The potential of combination therapy, such as antioxidants paired with anti-inflammatory drugs or neurotrophic factors, to improve therapeutic outcomes should also be explored in future clinical investigations [95]. Furthermore, the application of new biomarkers, including blood or inner ear fluid oxidative stress markers, can aid in patient stratification and therapy response monitoring [41]. The creation of uniform clinical trial procedures and outcome measures can make it easier to compare and synthesize data from many trials, which will ultimately result in evidence-based recommendations for the treatment of hearing loss [96]. Though there has been progress in our knowledge and management of oxidative stress-induced hearing loss, there are still a number of obstacles to overcome. Since the blood–labyrinth barrier prevents systemic medications from entering the cochlea, getting antioxidants to the inner ear is a significant difficulty [97]. Although local delivery techniques, like round window administration and intratympanic injections, have shown potential, they can be intrusive and necessitate repeated treatments [98]. To increase patient compliance and treatment effectiveness, non-invasive, sustained-release medication administration devices must be developed [99]. The variety of causes of hearing loss and the absence of particular diagnostic instruments to pinpoint hearing loss associated with oxidative stress present further difficulties [100]. The emergence of innovative diagnostic technologies, like genetic testing and imaging, can aid in customizing treatment plans according to the underlying cause of hearing loss [101]. Furthermore, research is required to determine the long-term safety and effectiveness of antioxidant treatments, especially in light of chronic use and possible drug interactions [102]. There are many prospects for more research in the area of oxidative stress and hearing loss. The creation of regenerative treatments, such as those based on stem cells, to replace damaged cochlear neurons and hair cells is one exciting field [103]. Regenerative methods, in conjunction with antioxidant therapy, may have a synergistic effect on hearing function restoration [104]. The use of gene therapy to alter the production of protective factors and antioxidant enzymes in the cochlea is an additional topic of research [73]. Personalized preventive and treatment techniques related to oxidative stress-associated hearing loss can be developed with the help of genetic risk factor identification [105]. Additionally, new paths for intervention may become available as a result of research on the gut–brain–ear axis and the function of the microbiota in regulating inflammation and oxidative stress in the auditory system [106]. Lastly, the combination of machine learning and big data analytics can aid in the discovery of new therapeutic targets and the optimization of treatment plans according to the unique characteristics of each patient [107,108].

## 10. Conclusions

This thorough analysis has brought to light the crucial part that oxidative stress plays in the pathophysiology of hearing loss and the promise that antioxidant-based treatments can play in both preventing and curing this crippling illness. The auditory system, and especially the cochlea, has special physiological and anatomical properties that make it extremely vulnerable to oxidative injury. Age-related, noise-induced, and ototoxic drug-induced hearing loss are among the types of hearing loss that have been linked to excessive reactive oxygen species (ROS) formation and the lowering of endogenous antioxidant defenses. Numerous pathways, including ischemia–reperfusion injury, inflammation, hair cell death, and mitochondrial dysfunction, are involved in the mechanisms behind oxidative stress-induced hearing loss. Preclinical and clinical research has demonstrated the potential of targeting these pathways with antioxidant therapies, such as pharmacological drugs, gene therapy, targeted delivery systems, and dietary antioxidants. Furthermore, alterations in lifestyle, such as lowering noise levels and adhering to a nutritious diet and regular exercise routine, might bolster general antioxidant defenses and lower the likelihood of hearing impairments. The review’s conclusions have significant ramifications for clinical practice when it comes to managing hearing loss. First off, measuring antioxidant status and oxidative stress markers can be useful diagnostic and prognosis markers for determining who is at risk for hearing loss and tracking how well a treatment is working. Second, including foods and supplements high in antioxidants in the diet may offer a secure and practical way to stop and lessen hearing loss brought on by oxidative stress. Third, pharmaceutical antioxidants, like coenzyme Q10 and N-acetylcysteine, may be used as adjuvant therapy for the treatment of hearing loss, especially when treating ototoxic drugs or exposure to noise. Additionally, more focused and effective methods of preventing oxidative damage to the auditory system may be available with the development of gene therapy techniques and tailored antioxidant delivery systems. Large-scale clinical studies and additional research will be necessary to validate these cutting-edge treatments before they can be used in clinical settings. Lastly, encouraging healthy lifestyles and putting noise reduction techniques into practice in both work and leisure environments can be crucial preventative measures against hearing loss brought on by oxidative stress. New preventive and therapeutic approaches have been made possible by the substantial advancements in our understanding of the role of oxidative stress in hearing loss in recent years. Nevertheless, a number of obstacles still need to be overcome, such as the requirement for more specialized diagnostic instruments, the improvement of medication delivery techniques, and the demonstration of antioxidant therapy’s long-term safety and effectiveness. Future investigations into the possibility of regenerative and customized therapy, as well as the intricate interactions between oxidative stress and other pathogenic pathways in hearing loss, should be the main priorities. To translate research discoveries into practical therapeutic solutions, a multidisciplinary strategy combining basic scientists, doctors, engineers, and industry partners is important. Through the advancement of knowledge on oxidative stress in the auditory system and the creation of novel therapeutic approaches, the lives of millions of people afflicted with hearing loss globally can be enhanced. In the end, preventing and treating oxidative stress-related hearing loss will call for an all-encompassing, integrative strategy that incorporates lifestyle changes, antioxidant therapies, and other therapeutic modalities.

## Figures and Tables

**Table 1:** Antioxidant strategies for the prevention and treatment of hearing loss.

Strategy	Study	Intervention	Model	Outcomes	References
Dietary antioxidants	Someya et al. (2010)	Mitochondrial-targeted vitamin C	Mice with age-related hearing loss	Reduced oxidative stress, prevented age-related hearing loss	[64]
	Heman-Ackah et al. (2010)	Oral combination antioxidant supplement	Guinea pigs exposed to noise	Reduced noise-induced hearing loss	[55]
	Gopinath et al. (2011)	Dietary intake of vitamins A, C, and E	Blue Mountains Hearing Study (human)	Higher intake associated with lower risk of hearing loss	[58]
	Kang et al. (2014)	Dietary intake of vitamin C and magnesium	Nurses’ Health Study II (human)	Higher intake associated with lower risk of hearing loss	[72]
Pharmacological agents	Kopke et al. (2007)	N-acetylcysteine (NAC)	Chinchillas exposed to noise	Reduced noise-induced hearing loss	[79]
	Feldman et al. (2007)	N-acetylcysteine (NAC)	Rats exposed to ototoxic drugs	Protected against ototoxic drug-induced hearing loss	[84]
	Someya et al. (2007)	Coenzyme Q10 (CoQ10)	Mice with age-related hearing loss	Attenuated age-related hearing loss	[85]
	Salami et al. (2010)	Coenzyme Q10 (CoQ10)	Human subjects with age-related hearing loss	Improved hearing function	[65]
	Campbell et al. (2007)	D-methionine	Chinchillas exposed to noise	Protected against noise-induced hearing loss	[66]
	Lynch et al. (2005)	Ebselen	Rats exposed to ototoxic drugs	Protected against ototoxic drug-induced hearing loss	[61]
Targeted delivery	Gao et al. (2018)	Resveratrol-loaded nanoparticles	Mice with age-related hearing loss	Enhanced efficacy in attenuating age-related hearing loss compared to systemic delivery	[86]
Gene therapy	Kawamoto et al. (2001)	Adenoviral-mediated catalase gene delivery	Guinea pigs with noise-induced hearing loss	Prevented noise-induced hair cell death and hearing loss	[73]
	Bao et al. (2005)	AAV-mediated superoxide dismutase gene delivery	Mice with age-related hearing loss	Attenuated age-related hearing loss	[87]

## References

[1] World Report on Hearing Consultato: 10 Giugno 2024. https://www.who.int/publications-detail-redirect/9789240020481.

[2] Ciorba A., Bianchini C., Pelucchi S., Pastore A. (2012). The impact of hearing loss on the quality of life of elderly adults. Clin. Interv. Aging.

[3] Lin F.R., Metter E.J., O’brien R.J., Resnick S.M., Zonderman A.B., Ferrucci L. (2011). Hearing Loss and Incident Dementia. Arch. Neurol..

[4] Fujimoto C., Yamasoba T. (2014). Oxidative stresses and mitochondrial dysfunction in age-related hearing loss. Oxidative Med. Cell. Longev..

[5] Pizzino G., Irrera N., Cucinotta M., Pallio G., Mannino F., Arcoraci V., Squadrito F., Altavilla D., Bitto A. (2017). Oxidative Stress: Harms and Benefits for Human Health. Oxid. Med. Cell. Longev..

[6] Liguori I., Russo G., Curcio F., Bulli G., Aran L., DELLA-Morte D., Gargiulo G., Testa G., Cacciatore F., Bonaduce D. (2018). Oxidative stress, aging, and diseases. Clin. Interv. Aging.

[7] Prasad K.N. (2017). Oxidative stress and pro-inflammatory cytokines may act as one of the signals for regulating microRNAs expression in Alzheimer’s disease. Mech. Ageing Dev..

[8] Reuter S., Gupta S.C., Chaturvedi M.M., Aggarwal B.B. (2010). Oxidative stress, inflammation, and cancer: How are they linked?. Free Radic. Biol. Med..

[9] Henderson D., Bielefeld E.C., Harris K.C., Hu B.H. (2006). The role of oxidative stress in noise-induced hearing loss. Ear Hear..

[10] Rybak L.P., Mukherjea D., Ramkumar V. (2019). Mechanisms of Cisplatin-Induced Ototoxicity and Prevention. Semin. Hear..

[11] Fetoni A.R., Paciello F., Rolesi R., Paludetti G., Troiani D. (2019). Targeting dysregulation of redox homeostasis in noise-induced hearing loss: Oxidative stress and ROS signaling. Free. Radic. Biol. Med..

[12] Raphael Y., Altschuler R.A. (2003). Structure and innervation of the cochlea. Brain Res. Bull..

[13] Rubel E.W., Furrer S.A., Stone J.S. (2013). A brief history of hair cell regeneration research and speculations on the future. Hear. Res..

[14] Clerici W.J., Yang L. (1996). Direct effects of intraperilymphatic reactive oxygen species generation on cochlear function. Hear. Res..

[15] Kopke R., Allen K.A., Henderson D., Hoffer M., Frenz D., VAN DE Water T. (1999). A radical demise: Toxins and trauma share common pathways in hair cell death. Ann. N. Y. Acad. Sci..

[16] Cag Y., Al Madadha M.E., Ankarali H., Cag Y., Onder K.D., Seremet-Keskin A., Kizilates F., Čivljak R., Shehata G., Alay H. (2022). Vaccine hesitancy and refusal among parents: An international ID-IRI survey. J. Infect. Dev. Ctries..

[17] Neri S., Signorelli S., Pulvirenti D., Mauceri B., Cilio D., Bordonaro F., Abate G., Interlandi D., Misseri M., Ignaccolo L. (2006). Oxidative stress, nitric oxide, endothelial dysfunction and tinnitus. Free. Radic. Res..

[18] Sies H. (2015). Oxidative stress: A concept in redox biology and medicine. Redox Biol..

[19] Phaniendra A., Jestadi D.B., Periyasamy L. (2015). Free radicals: Properties, sources, targets, and their implication in various diseases. Indian J. Clin. Biochem..

[20] Di Meo S., Reed T.T., Venditti P., Victor V.M. (2016). Role of ROS and RNS Sources in Physiological and Pathological Conditions. Oxid. Med. Cell. Longev..

[21] Böttger E.C., Schacht J. (2013). The mitochondrion: A perpetrator of acquired hearing loss. Hear. Res..

[22] Yang Y., Chen X., Tian C., Fan B., An X., Liu Z., Li Q., Mi W., Lin Y., Zha D. (2024). Gene expression analysis of oxidative stress-related genes in the apical, middle, and basal turns of the cochlea. Gene Expr. Patterns.

[23] Kamogashira T., Fujimoto C., Yamasoba T. (2015). Reactive Oxygen Species, Apoptosis, and Mitochondrial Dysfunction in Hearing Loss. BioMed Res. Int..

[24] Maulucci G., Troiani D., Eramo S.L.M., Paciello F., Podda M.V., Paludetti G., Papi M., Maiorana A., Palmieri V., De Spirito M. (2014). Time evolution of noise induced oxidation in outer hair cells: Role of NAD(P)H and plasma membrane fluidity. Biochim. Biophys. Acta (BBA)-Gen. Subj..

[25] Jiang M., Karasawa T., Steyger P.S. (2017). Aminoglycoside-Induced Cochleotoxicity: A Review. Front. Cell. Neurosci..

[26] Sheth S., Mukherjea D., Rybak L.P., Ramkumar V. (2017). Mechanisms of Cisplatin-Induced Ototoxicity and Otoprotection. Front. Cell Neurosci..

[27] Fetoni A.R., Picciotti P.M., Paludetti G., Troiani D. (2011). Pathogenesis of presbycusis in animal models: A review. Exp. Gerontol..

[28] Poirrier A.L., Pincemail J., Ackerveken P.V.D., Lefebvre P.P., Malgrange B. (2010). Oxidative stress in the cochlea: An update. Curr. Med. Chem..

[29] Rybak L.P., Husain K., Morris C., Whitworth C., Somani S. (2000). Effect of protective agents against cisplatin ototoxicity. Otol. Neurotol..

[30] Fetoni A., Paciello F., Rolesi R., Eramo S., Mancuso C., Troiani D., Paludetti G. (2015). Rosmarinic acid up-regulates the noise-activated Nrf2/HO-1 pathway and protects against noise-induced injury in rat cochlea. Free. Radic. Biol. Med..

[31] Wang J., Puel J.-L. (2020). Presbycusis: An Update on Cochlear Mechanisms and Therapies. J. Clin. Med..

[32] Kalinec G.M., Lomberk G., Urrutia R.A., Kalinec F. (2017). Resolution of Cochlear Inflammation: Novel Target for Preventing or Ameliorating Drug-, Noise- and Age-related Hearing Loss. Front. Cell. Neurosci..

[33] Shi X. (2016). Pathophysiology of the cochlear intrastrial fluid-blood barrier (review). Hear. Res..

[34] Guo J., Chai R., Li H., Sun S. (2019). Protection of Hair Cells from Ototoxic Drug-Induced Hearing Loss. Adv. Exp. Med. Biol..

[35] Bai U., Seidman M.D., Hinojosa R., Quirk W.S. (1997). Mitochondrial DNA deletions associated with aging and possibly presbycusis: A human archival temporal bone study. Otol. Neurotol..

[36] Markaryan A., Nelson E.G., Hinojosa R. (2009). Quantification of the mitochondrial DNA common deletion in presbycusis. Laryngoscope.

[37] Wong A.C.Y., Ryan A.F. (2015). Mechanisms of sensorineural cell damage, death and survival in the cochlea. Front. Aging Neurosci..

[38] Vandenabeele P., Galluzzi L., Vanden Berghe T.V., Kroemer G. (2010). Molecular mechanisms of necroptosis: An ordered cellular explosion. Nat. Rev. Mol. Cell Biol..

[39] Elmore S. (2007). Apoptosis: A review of programmed cell death. Toxicol. Pathol..

[40] Orrenius S., Gogvadze V., Zhivotovsky B. (2007). Mitochondrial Oxidative Stress: Implications for Cell Death. Annu. Rev. Pharmacol. Toxicol..

[41] Rybak L.P., Mukherjea D., Jajoo S., Ramkumar V. (2009). Cisplatin Ototoxicity and Protection: Clinical and Experimental Studies. Tohoku J. Exp. Med..

[42] Wei L., Ding D., Salvi R. (2010). Salicylate-induced degeneration of cochlea spiral ganglion neurons-apoptosis signaling. Neuroscience.

[43] Sha S.-H., Schacht J. (2017). Emerging therapeutic interventions against noise-induced hearing loss. Expert Opin. Investig. Drugs.

[44] Tan W.J.T., Thorne P.R., Vlajkovic S.M. (2016). Characterisation of cochlear inflammation in mice following acute and chronic noise exposure. Histochem..

[45] Okano T., Nakagawa T., Kita T., Kada S., Yoshimoto M., Nakahata T., Ito J. (2008). Bone marrow-derived cells expressing Iba1 are constitutively present as resident tissue macrophages in the mouse cochlea. J. Neurosci. Res..

[46] Fujioka M., Kanzaki S., Okano H.J., Masuda M., Ogawa K., Okano H. (2006). Proinflammatory cytokines expression in noise-induced damaged cochlea. J. Neurosci. Res..

[47] Watson N., Ding B., Zhu X., Frisina R.D. (2017). Chronic inflammation–inflammaging–in the ageing cochlea: A novel target for future presbycusis therapy. Ageing Res. Rev..

[48] Tabuchi K., Nishimura B., Tanaka S., Hayashi K., Hirose Y., Hara A. (2010). Ischemia-reperfusion injury of the cochlea: Pharmacological strategies for cochlear protection and implications of glutamate and reactive oxygen species. Curr. Neuropharmacol..

[49] Tsutsui H., Kinugawa S., Matsushima S. (2011). Oxidative stress and heart failure. Am. J. Physiol.-Heart Circ. Physiol..

[50] Jarrard C.P., Nagel M.J., Stray-Gundersen S., Tanaka H., Lalande S. (2021). Hypoxic preconditioning attenuates ischemia-reperfusion injury in young healthy adults. J. Appl. Physiol..

[51] Kaya H., Koç A.K., Sayın İ., Güneş S., Altıntaş A., Yeğin Y., Kayhan F.T. (2015). Vitamins A, C, and E and selenium in the treatment of idiopathic sudden sensorineural hearing loss. Eur. Arch. Oto-Rhino-Laryngol..

[52] Prasad K.N., Bondy S.C. (2020). Increased oxidative stress, inflammation, and glutamate: Potential preventive and therapeutic targets for hearing disorders. Mech. Ageing Dev..

[53] Seidman M.D., Babu S. (2003). Alternative medications and other treatments for tinnitus: Facts from fiction. Otolaryngol. Clin. N. Am..

[54] Schiavone S., Jaquet V., Trabace L., Krause K.-H. (2013). Severe life stress and oxidative stress in the brain: From animal models to human pathology. Antioxid Redox Signal..

[55] Ackah S.E.H., Juhn S.K., Huang T.C., Wiedmann T.S. (2010). A combination antioxidant therapy prevents age-related hearing loss in C57BL/6 mice. Otolaryngol. Neck Surg..

[56] Leprell C., Hughes L.F., Miller J.M. (2007). Free radical scavengers vitamins A, C, and E plus magnesium reduce noise trauma. Free Radic. Biol. Med..

[57] Delhez A., Lefebvre P., Péqueux C., Malgrange B., Delacroix L. (2020). Auditory function and dysfunction: Estrogen makes a difference. Cell. Mol. Life Sci..

[58] Gopinath B., Flood V.M., McMahon C.M., Burlutsky G., Spankovich C., Hood L.J., Mitchell P. (2011). Dietary antioxidant intake is associated with the prevalence but not incidence of age-related hearing loss. J. Nutr. Health Aging.

[59] Noack V., Pak K., Jalota R., Kurabi A., Ryan A.F. (2017). An Antioxidant Screen Identifies Candidates for Protection of Cochlear Hair Cells from Gentamicin Toxicity. Front. Cell. Neurosci..

[60] Oishi N., Schacht J. (2011). Emerging treatments for noise-induced hearing loss. Expert Opin. Emerg. Drugs.

[61] Lynch E.D., Kil J. (2005). Compounds for the prevention and treatment of noise-induced hearing loss. Drug Discov. Today.

[62] Kopke R., Bielefeld E., Liu J., Zheng J., Jackson R., Henderson D., Coleman J.K.M. (2005). Prevention of impulse noise-induced hearing loss with antioxidants. Acta Oto-Laryngol..

[63] Kopke R.D., Coleman J.K.M., Liu J., Campbell K.C.M., Riffenburgh R.H., MC Usa R.D.K.C. (2002). Enhancing intrinsic cochlear stress defenses to reduce noise-induced hearing loss. Laryngoscope.

[64] Someya S., Xu J., Kondo K., Ding D., Salvi R.J., Yamasoba T., Rabinovitch P.S., Weindruch R., Leeuwenburgh C., Tanokura M. (2009). Age-related hearing loss in C57BL/6J mice is mediated by Bak-dependent mitochondrial apoptosis. Proc. Natl. Acad. Sci. USA.

[65] Salami A., Mora R., Dellepiane M., Manini G., Santomauro V., Barettini L., Guastini L. (2010). Water-soluble coenzyme Q10 formulation (Q-TER^®^) in the treatment of presbycusis. Acta Oto-Laryngol..

[66] Campbell K.C., Meech R.P., Klemens J.J., Gerberi M.T., Dyrstad S.S., Larsen D.L., Mitchell D.L., El-Azizi M., Verhulst S.J., Hughes L.F. (2007). Prevention of noise- and drug-induced hearing loss with d-methionine. Hear. Res..

[67] Lynch E.D., Gu R., Pierce C., Kil J. (2004). Ebselen-Mediated protection from single and repeated noise exposure in rat. Laryngoscope.

[68] Hahn H., Kammerer B., DiMauro A., Salt A.N., Plontke S.K. (2006). Cochlear microdialysis for quantification of dexamethasone and fluorescein entry into scala tympani during round window administration. Hear. Res..

[69] Buckiová D., Ranjan S., Newman T.A., Johnston A.H., Sood R., Kinnunen P.K., Popelář J., Chumak T., Syka J. (2012). Minimally invasive drug delivery to the cochlea through application of nanoparticles to the round window membrane. Nanomedicine.

[70] Zhang Y., Zhang W., Löbler M., Schmitz K.-P., Saulnier P., Perrier T., Pyykkö I., Zou J. (2011). Inner ear biocompatibility of lipid nanocapsules after round window membrane application. Int. J. Pharm..

[71] Natarajan N., Batts S., Stankovic K.M. (2023). Noise-Induced Hearing Loss. J. Clin. Med..

[72] Pharaoh G., Kamat V., Kannan S., Stuppard R.S., Whitson J., Martín-Pérez M., Qian W.-J., MacCoss M.J., Villén J., Rabinovitch P. (2023). The mitochondrially targeted peptide elamipretide (SS-31) improves ADP sensitivity in aged mitochondria by increasing uptake through the adenine nucleotide translocator (ANT). GeroScience.

[73] Kawamoto K., Sha S.-H., Minoda R., Izumikawa M., Kuriyama H., Schacht J., Raphael Y. (2004). Antioxidant gene therapy can protect hearing and hair cells from ototoxicity. Mol. Ther..

[74] Pfannenstiel S.C., Praetorius M., Plinkert P.K., Brough D.E., Staecker H. (2009). Bcl-2 Gene Therapy prevents aminoglycoside-induced degeneration of auditory and vestibular hair cells. Audiol. Neurotol..

[75] Abraham N.G., Asija A., Drummond G., Peterson S. (2007). Heme oxygenase-1 gene therapy: Recent advances and therapeutic applications. Curr. Gene Ther..

[76] Franzé A., Sequino L., Saulino C., Attanasio G., Marciano E. (2003). Effect over time of allopurinol on noise-induced hearing loss in guinea pigs: Efecto en el tiempo del alopurinol sobre la hipoacusia inducida por ruido en cobayos. Int. J. Audiol..

[77] Yang Y., Song H.L., Zhang W., Wu B.J., Fu N.N., Dong C., Shen Z.Y. (2016). Heme oxygenase-1-transduced bone marrow mesenchymal stem cells in reducing acute rejection and improving small bowel transplantation outcomes in rats. Stem Cell Res. Ther..

[78] Mener D.J., Betz J., Yaffe K., Harris T.B., Helzner E.P., Satterfield S., Houston D.K., Strotmeyer E.S., Pratt S.R., Simonsick E.M. (2016). Apolipoprotein E Allele and Hearing Thresholds in Older Adults. Am. J. Alzheimer’s Dis. Other Dement..

[79] Bielefeld E.C., Kopke R.D., Jackson R.L., Coleman J.K., Liu J., Henderson D. (2007). Noise protection with N-acetyl-l-cysteine (NAC) using a variety of noise exposures, NAC doses, and routes of administration. Acta Oto-Laryngol..

[80] Curhan S.G., Eavey R., Wang M., Stampfer M.J., Curhan G.C. (2013). Body mass index, waist circumference, physical activity, and risk of hearing loss in women. Am. J. Med..

[81] Itoh A., Nakashima T., Arao H., Wakai K., Tamakoshi A., Kawamura T., Ohno Y. (2001). Smoking and drinking habits as risk factors for hearing loss in the elderly: Epidemiological study of subjects undergoing routine health checks in Aichi, Japan. Public Health.

[82] Helzner E.P., Contrera K.J. (2016). Type 2 Diabetes and Hearing Impairment. Curr. Diabetes Rep..

[83] Frisina S.T., Mapes F., Kim S., Frisina D.R., Frisina R.D. (2006). Characterization of hearing loss in aged type II diabetics. Hear. Res..

[84] Feldman L., Efrati S., Eviatar E., Abramsohn R., Yarovoy I., Gersch E., Averbukh Z., Weissgarten J. (2007). Gentamicin-induced ototoxicity in hemodialysis patients is ameliorated by N-acetylcysteine. Kidney Int..

[85] Someya S., Yamasoba T., Kujoth G.C., Pugh T.D., Weindruch R., Tanokura M., Prolla T.A. (2008). The role of mtDNA mutations in the pathogenesis of age-related hearing loss in mice carrying a mutator DNA polymerase γ. Neurobiol. Aging.

[86] Cervantes B., Arana L., Murillo-Cuesta S., Bruno M., Alkorta I., Varela-Nieto I. (2019). Solid Lipid Nanoparticles Loaded with Glucocorticoids Protect Auditory Cells from Cisplatin-Induced Ototoxicity. J. Clin. Med..

[87] Bao J., Lei D., Du Y., Ohlemiller K.K., Beaudet A.L., Role L.W. (2005). Requirement of nicotinic acetylcholine receptor subunit β2 in the maintenance of spiral ganglion neurons during aging. J. Neurosci..

[88] Ohlemiller K.K., Wright J.S., Dugan L.L. (1999). Early elevation of cochlear reactive oxygen species following noise exposure. Audiol. Neurotol..

[89] Wangemann P. (2002). K+ cycling and the endocochlear potential. Hear. Res..

[90] Ottonelli I., Bighinati A., Adani E., Loll F., Caraffi R., Vandelli M.A., Boury F., Tosi G., Duskey J.T., Marigo V. (2022). Optimization of an Injectable Hydrogel Depot System for the Controlled Release of Retinal-Targeted Hybrid Nanoparticles. Pharmaceutics.

[91] Waldhaus J., Durruthy-Durruthy R., Heller S. (2015). Quantitative High-Resolution Cellular Map of the Organ of Corti. Cell Rep..

[92] Liu W., Xu X., Fan Z., Sun G., Han Y., Zhang D., Xu L., Wang M., Wang X., Zhang S. (2019). Wnt Signaling Activates TP53-Induced Glycolysis and Apoptosis Regulator and Protects Against Cisplatin-Induced Spiral Ganglion Neuron Damage in the Mouse Cochlea. Antioxid. Redox Signal..

[93] Bowl M.R., Dawson S.J. (2019). Age-Related Hearing Loss. Cold Spring Harb. Perspect. Med..

[94] Kopke R.D., Jackson R.L., Coleman J.K., Liu J., Bielefeld E.C., Balough B.J. (2007). NAC for noise: From the bench top to the clinic. Hear. Res..

[95] Sly D.J., Campbell L., Uschakov A., Saief S.T., Lam M., O’leary S.J. (2016). Applying Neurotrophins to the Round Window Rescues Auditory Function and Reduces Inner Hair Cell Synaptopathy After Noise-induced Hearing Loss. Otol. Neurotol..

[96] Ding D., Jiang H., Chen G.-D., Longo-Guess C., Muthaiah V.P.K., Tian C., Sheppard A., Salvi R., Johnson K.R. (2016). N-acetyl-cysteine prevents age-related hearing loss and the progressive loss of inner hair cells in γ-glutamyl transferase 1 deficient mice. Aging.

[97] Kujawa S.G., Liberman M.C. (2019). Translating animal models to human therapeutics in noise-induced and age-related hearing loss. Hear. Res..

[98] Salt A.N., Hirose K. (2018). Communication pathways to and from the inner ear and their contributions to drug delivery. Hear. Res..

[99] Li L., Chao T., Brant J., O’Malley B., Tsourkas A., Li D. (2017). Advances in nano-based inner ear delivery systems for the treatment of sensorineural hearing loss. Adv. Drug Deliv. Rev..

[100] El Kechai N., Agnely F., Mamelle E., Nguyen Y., Ferrary E., Bochot A. (2015). Recent advances in local drug delivery to the inner ear. Int. J. Pharm..

[101] Cruickshanks K.J., Nondahl D.M., Tweed T.S., Wiley T.L., Klein B.E., Klein R., Chappell R., Dalton D.S., Nash S.D. (2010). Education, occupation, noise exposure history and the 10-yr cumulative incidence of hearing impairment in older adults. Hear. Res..

[102] Lanvers-Kaminsky C., Zehnhoff-Dinnesen A.A., Parfitt R., Ciarimboli G. (2017). Drug-induced ototoxicity: Mechanisms, Pharmacogenetics, and protective strategies. Clin. Pharmacol. Ther..

[103] Kishimoto-Urata M., Urata S., Fujimoto C., Yamasoba T. (2022). Role of Oxidative Stress and Antioxidants in Acquired Inner Ear Disorders. Antioxidants.

[104] Zhang S., Qiang R., Dong Y., Zhang Y., Chen Y., Zhou H., Chai R. (2020). Hair cell regeneration from inner ear progenitors in the mammalian cochlea. Am. J. Stem Cells.

[105] McFadden S.L., Woo J.M., Michalak N., Ding D. (2005). Dietary vitamin C supplementation reduces noise-induced hearing loss in guinea pigs. Hear. Res..

[106] Delmaghani S., El-Amraoui A. (2020). Inner Ear Gene Therapies Take Off: Current Promises and Future Challenges. J. Clin. Med..

[107] Kunst C., Schmid S., Michalski M., Tümen D., Buttenschön J., Müller M., Gülow K. (2023). The Influence of Gut Microbiota on Oxidative Stress and the Immune System. Biomedicines.

[108] Wimalarathna H., Ankmnal-Veeranna S., Duong M., Allan C., Agrawal S.K., Allen P., Ladak H.M. (2023). Using machine learning to assist auditory processing evaluation. Front. Audiol. Otol..

